# Massive Intra-Abdominal Imatinib-Resistant Gastrointestinal Stromal Tumor in a 21-Year-Old Male

**DOI:** 10.1155/2013/373981

**Published:** 2013-08-04

**Authors:** Ann Falor, Amanda K. Arrington, Carrie Luu, Hans F. Schoellhammer, Michelle Ko, Warren Chow, Massimo D'Apuzzo, Jinha Park, Joseph Kim

**Affiliations:** ^1^Department of Surgery, Harbor-UCLA Medical Center, 1000 West Carson Street, Torrance, CA 90509, USA; ^2^Division of Surgical Oncology, City of Hope Comprehensive Cancer Center, City of Hope, 1500 East Duarte Road, Duarte, CA 91010, USA; ^3^Department of Medical Oncology & Therapeutics Research, City of Hope Comprehensive Cancer Center, 1500 East Duarte Road, Duarte, CA 91010, USA; ^4^Department of Pathology, City of Hope Comprehensive Cancer Center, 1500 East Duarte Road, Duarte, CA 91010, USA; ^5^Department of Radiology, City of Hope Comprehensive Cancer Center, 1500 East Duarte Road, Duarte, CA 91010, USA

## Abstract

Gastrointestinal stromal tumors (GISTs) in adolescence are far less common than adult GISTs and have varied GIST genotypes that present diagnostic and therapeutic challenges. Here, we discuss a 21-year-old male with diagnosis of unresectable, imatinib-resistant GIST. At initial evaluation, a neoadjuvant treatment approach was recommended. As such, the patient received imatinib over the course of one year. Unfortunately, the GIST increased in size, and a subsequent attempt at surgical resection was aborted fearing infiltration of major vascular structures. The patient was then referred to our institution, at which time imatinib therapy was discontinued. Surgical intervention was again considered and the patient underwent successful resection of massive intra-abdominal GIST with total gastrectomy and Roux-en-Y esophagojejunostomy. Since pediatric GISTs are typically resistant to imatinib, we performed genotype analysis of the operative specimen that revealed KIT mutations associated with imatinib sensitivity and resistance. Given the sequencing data and operative findings, the patient was started postoperatively on sunitinib. This case illustrates the importance of understanding both adult and pediatric GISTs when implementing appropriate treatment regimens. Since the genotype of GISTs dictates phenotypic behavior, mutational analysis is an important component of care especially for adolescents whose disease may mirror the pediatric or adult population.

## 1. Introduction

Gastrointestinal stromal tumors (GISTs) represent the most common mesenchymal tumor of the gastrointestinal tract in the adult population, with a reported incidence rate of 6.8 cases per million [1]. These tumors are commonly diagnosed in the stomach or small intestine, and the median age at diagnosis is 60 years [2]. GISTs are far less common in children, and pediatric GISTs appear to be different in relation to the disease observed in adults. Moreover, variations of GIST genotype in the pediatric population present diagnostic and therapeutic challenges. Here, we discuss the presentation and management of a GIST in an adolescent male to highlight the difficulties in managing this rare disease. 

## 2. Case Report

A 21-year-old African-American male was referred to our tertiary care cancer center with the diagnosis of unresectable, imatinib-resistant GIST. Institutional review board approval was obtained to review his clinical course. He was initially evaluated at another institution approximately one year earlier for a several-month history of gastroesophageal reflux disease, weight loss, and increasing abdominal distension. At the time of this initial presentation, the patient had these nonspecific abdominal complaints but was otherwise healthy. He underwent esophagogastroduodenoscopy (EGD), which demonstrated a large ulcerated tumor near the gastroesophageal junction. Biopsy of the mass revealed a spindle cell lesion and CD117 (i.e., c-KIT) immunohistochemistry (IHC) staining confirmed the diagnosis of GIST. Computed tomography (CT) imaging demonstrated an 11 × 10 × 8 cm lesion arising from the stomach and no other lesions elsewhere. Due to uncertainty about upfront surgical resectability, the patient was started on imatinib (400 mg/day), a tyrosine kinase inhibitor (TKI) that blocks the constitutively active receptor tyrosine kinase c-KIT, to induce adequate tumor response for potential surgical resection with negative margins [3–5].

**Figure 1:**
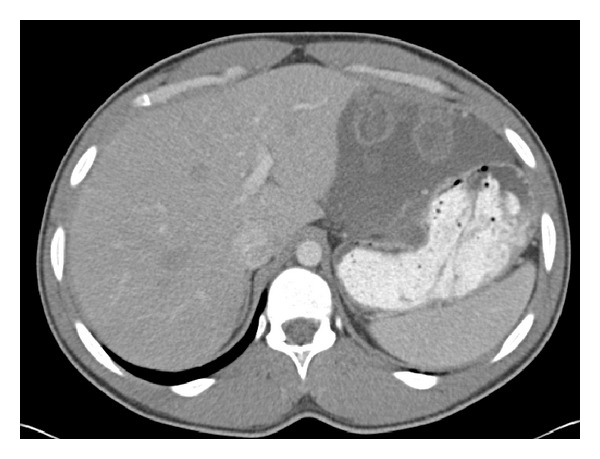
Computed tomography (CT) image demonstrating the appearance of gastric GIST after seven months of treatment with imatinib 400 mg per day; the tumor is unchanged in size compared to initial presentation (initial CT not shown).

**Figure 2:**
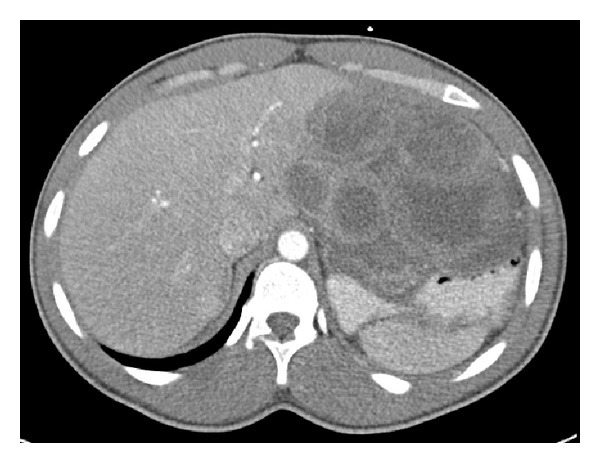
CT image showing increase in tumor size four months after doubling the dosage of imatinib to 800 mg per day.

On follow-up CT scan nearly seven months later (Figure 1), there was no evidence of decreased tumor dimensions. Consequently, the dosage of imatinib was doubled to 800 mg daily. Four months after this change in dosage, the patient complained of unrelenting abdominal pain necessitating CT imaging (Figure 2) which revealed that the GIST had increased in size to 15 × 15 × 12 cm. This extent of disease raised new concerns for tumor invasion of the celiac axis. With failure of the downstaging approach, the patient underwent attempt at salvage surgical resection approximately one year after initial diagnosis. During diagnostic laparoscopy the tumor was considered to be infiltrative and unresectable, and a feeding jejunostomy catheter was placed for palliation. The patient subsequently sought further care at our tertiary care cancer center. 

On our initial examination, the patient had a largely protuberant abdomen with a mass palpable from the costal margin to the pelvic brim. He was no longer able to eat and repeat EGD demonstrated a cystic/solid gastric mass arising from the level of the gastric cardia, leading to extensive extrinsic compression of the entire stomach. The celiac axis could not be visualized on endoscopic ultrasound. Pathologic review of the original biopsy specimen revealed a moderately cellular spindle cell lesion with minimal atypia and no mitosis. IHC revealed that the cells stained strongly positive for CD34 and CD117, again confirming the diagnosis of GIST. Based on our experience with GISTs, we theorized that this patient's increased extent of disease was producing mass effect and compression of surrounding organs rather than infiltrating the surrounding tissues; therefore, our surgical team recommended operative exploration. 

**Figure 3:**
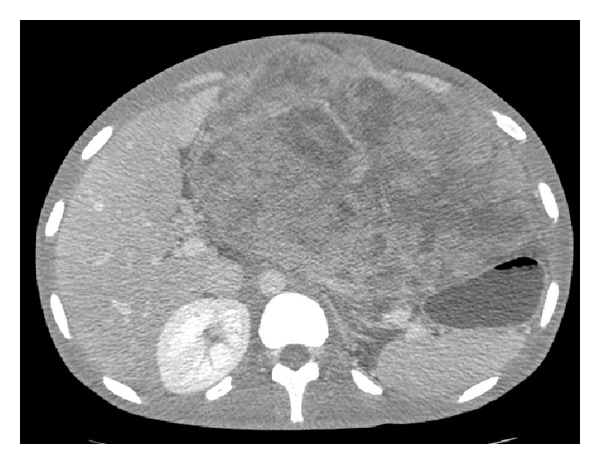
Preoperative CT image revealing a single massive tumor (30 × 21 × 13 cm).

**Figure 4:**
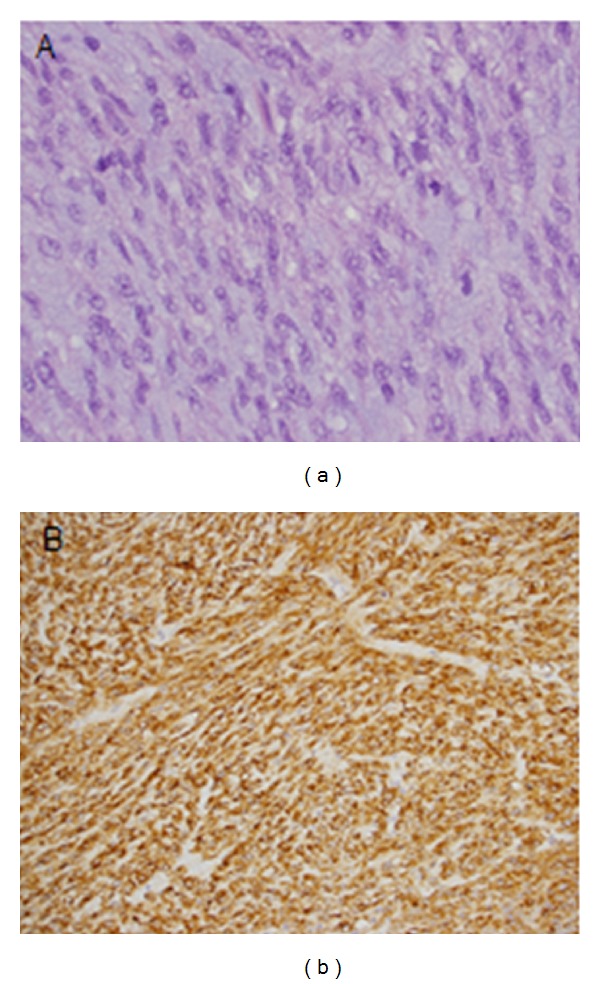
(a) Photomicrograph of the resected specimen stained with H + E showing features of high grade GIST with mitotically active spindle and epithelioid cells involving the gastric and perigastric adipose tissue (60x magnification). (b) IHC staining demonstrating strong diffuse staining for CD117 (20x magnification).

More than one year following his initial diagnosis, the patient underwent resection of a massive intra-abdominal GIST with total gastrectomy and Roux-en-Y esophagojejunostomy. On our preoperative imaging the GIST measured 30 × 21 × 13 cm (Figure 3), and the resected abdominal specimen revealed two masses on gross examination measuring 30 × 19 × 13 cm and 8.5 × 6 × 5.5 cm. The neoplasm showed features of high-grade GIST with mitotically active spindle and epithelioid cells involving the gastric and perigastric adipose tissue (Figure 4(a), 60x magnification), and IHC staining demonstrated strong diffuse staining for CD117 (Figure 4(b), 20x magnification). Despite preoperative imaging suggesting compression of the celiac axis, the mass was easily lifted off the major vessels. Unfortunately, a small isolated pelvic nodule was discovered and found to be metastatic disease, yielding a final pathologic stage of ypT4N0M1. The patient tolerated the procedure without incident and was discharged home on postoperative day 9.

At our institution, genetic testing of the c-KIT gene includes selective assays of exons 8, 9, 11, 13, 17, and 18. These exons are amplified by polymerase chain reaction (PCR) and then undergo Sanger sequencing. Any mutations found are then confirmed via a second independent amplification. Genotype analysis of KIT in the operative specimen revealed an exon 11 deletion in codons 557–559, which is known to confer imatinib sensitivity [6]. An exon 17 mutation in codon 820 of the kinase activating loop, which is associated with imatinib resistance, was also identified [6–8]. Given the sequencing data and presence of metastatic disease, the patient was started on sunitinib 50 mg daily given for cycles of four weeks at a time, with a two-week break between cycles. Sunitinib is a multitarget TKI against vascular endothelial growth factor receptor (VEGFR), platelet-derived growth factor receptor (PDGFR), and c-KIT that is effective in eliciting tumor response and disease stabilization in imatinib-resistant GISTs [9]. However, the patient progressed on sunitinib, and subsequently sorafenib 400 mg twice daily given continuously was initiated. Sorafenib is a multiple kinase inhibitor that targets VEGFR, PDGFR, RET, and c-KIT [10]. Sorafenib has been shown to prevent disease progression in patients with imatinib and sunitinib refractory GIST [11]. 

## 3. Discussion

**Figure 5:**
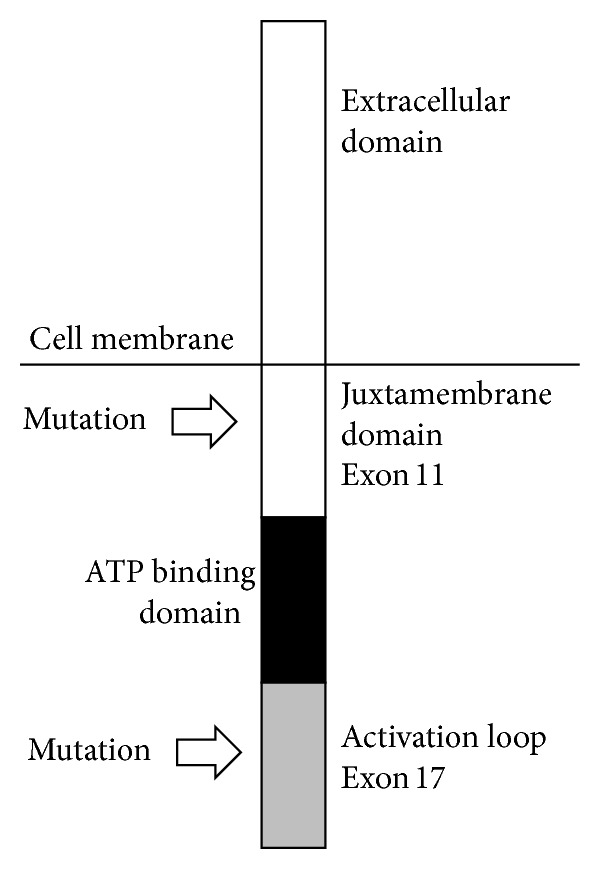
Schematic diagram of the intra- and extracellular KIT protein domains coded for by various exons as well as the mutations in exons 11 and 17 seen in our patient.

GISTs frequently harbor gain-of-function mutations in the KIT or PDGFRA protooncogenes, which are detected in 90% of adult GIST patients [12, 13]. While KIT mutations are more common in exon 11, mutations have also been detected in exons 9, 13, and 17 [8]. Targeted inhibition of these receptor tyrosine kinases has been developed, but the exact location of the genetic alterations predicts response to the targeted agents. Specifically, KIT mutations located on exon 11 are associated with sensitivity to imatinib therapy, whereas mutations on exons 9, 13, and 17 are more likely to exhibit imatinib resistance [7, 12, 14, 15]. KIT mutations may also have prognostic significance, and patients with exon 11 mutations appear to have improved outcomes compared to patients harboring mutations on exons 9 or 13 [12, 16, 17]. Exon 11 of the KIT gene codes for the juxtamembrane domain of the KIT protein, and exon 17 codes for the activation loop of the protein, which is intracellular. Approximately 70% of primary KIT mutations are in exon 11, while only 1% of primary mutations are in exon 17 [18]. Secondary mutations of exon 17 also may occur, which can lead to resistance to treatment with sunitinib but not sorafenib, as was seen with our patient [18].  Figure 5 demonstrates a schematic representation of the various domains of the KIT protein, the exons that code for these domains, and the sites of codon mutations seen in our patient.

GISTs in the pediatric population are rare with a reported incidence of 0.5–2.7% and a median age at presentation of 13 years [13, 19]. Similar to adults, the location of disease is most often in the stomach [19]. Pediatric GISTs can occur sporadically or as part of familial syndromes, such as Carney-Stratakis syndrome, which is secondary to mutations in succinate dehydrogenase (SDH) which predisposes affected individuals to GISTs and paragangliomas [13]. The biology of disease differs greatly between pediatric and adult patients since most pediatric GISTs harbor wild-type KIT or PDGFRA, thus lacking the activating mutations that confer sensitivity to imatinib. Therefore, imatinib is often ineffective in pediatric GISTs [20]. Interestingly, mutations in SDH have been detected in nearly 12% of pediatric patients with nonfamilial GIST and wild-type KIT [13]. Though germline testing for SDH mutations have been advocated in pediatric GISTs with wild-type KIT, the treatment of SDH mutated GIST is currently the same as non-SDH mutated GIST, and the clinical significance of SDH mutations remains unclear [13, 19]. 

Surgical resection with clear microscopic margins is the gold standard treatment for both pediatric and adult GISTs. In our patient, imatinib therapy was used preoperatively in an attempt to decrease disease burden and minimize the extent of surgical intervention [21, 22]. Ultimately, this neoadjuvant approach failed, and the final surgical procedure carried far higher potential for morbidity and mortality than with the original presenting disease. DNA sequencing of this final operative specimen revealed both imatinib-sensitive and imatinib-resistant features, unlike typical wild-type KIT GISTs in pediatric patients. We hypothesize that the KIT exon 11 deletion was present *de novo* prior to initiation of imatinib therapy, and that resistance may be secondary to the subsequent development of the exon 17 mutation, a phenomenon that has been described in adult patients [6, 7]. However, it is still feasible that both exons 11 and 17 mutations were present *de novo*, a condition that would have altered the management strategy leading to sunitinib as the initial neoadjuvant agent. Furthermore, knowledge of the mutations beforehand may have provided the rationale to change to sunitinib or plan for surgery earlier in the disease course, when the patient initially failed to demonstrate treatment response to imatinib.

 Upon recovering from surgery, our patient was started on sunitinib. Unlike imatinib, sunitinib has been discovered to be effective in the pediatric GIST population [6]. The genetic makeup of GISTs dictates biologic behavior, and it is of paramount importance that clinicians have a clear understanding of the differences between adult and pediatric GISTs. Our patient, although 21 years old at the time of his definitive operation, had experienced symptoms beginning in his teenage years. Despite his age, the genotype of his GIST was more akin to adult patients with a gain-of-function KIT mutation. Nevertheless, knowledge of the imatinib resistant genotype may have precluded imatinib therapy altogether. Although it may not be necessary to perform mutational analysis of KIT and PDGFRA prior to initiating therapy in all pediatric GIST patients, upfront DNA sequencing may benefit the adolescent patient whose disease may mirror either the pediatric or adult population. This case highlights the need for knowledge of differences between adult and pediatric GISTs and the need for specialized care of this rare disease.
